# Monkeypox: An Unfamiliar Virus—Clinical and Epidemiological Characteristics, Diagnosis, and Treatment with Special Emphasis on Oral Health

**DOI:** 10.3390/diagnostics12112749

**Published:** 2022-11-10

**Authors:** Visha Shailesh Pandya, Vini Mehta, Mohammas Miraj, Sharifa M. Alasiry, Wdad Alanazy, Tintu Thomas Uthup, Riyaz Ahamed Shaik, Cesare D’Amico, Maura Mancini, Francesca Gorassini, Luca Fiorillo, Aida Meto

**Affiliations:** 1 Department of Public Health Dentistry, Vaidik Dental College & Research Centre, Dadra and Nagar Haveli and Daman and Diu 396210, India; 2 Department of Public Health Dentistry, Dr. D.Y. Patil Dental College and Hospital, Dr. D.Y. Patil Vidyapeeth, Pimpri, Pune 411018, India; 3 Department of Physical Therapy and Rehabilitation, College of Applied Medical Sciences, Majmaah University, Al-Majmaah 11952, Saudi Arabia; 4 Department of Nursing, College of Applied Medical Sciences, Majmaah University, Al-Majmaah 11952, Saudi Arabia; 5 Department of Maternity Nursing, Faculty of Nursing, College of Applied Medical Sciences, Majmaah University, Al-Majmaah 11952, Saudi Arabia; 6 Department of Public Health, College of Health Sciences, Saudi Electronic University, Riyadh 13316, Saudi Arabia; 7 Department of Family and Community Medicine, College of Medicine, Majmaah University, Al-Majmaah 11952, Saudi Arabia; 8 Department of Biomedical and Dental Sciences, Morphological and Functional Images, University of Messina, 98100 Messina, Italy; 9 Unit of Ophthalmology, Department of Biomedical and Dental Sciences, Morphological and Functional Images, University of Messina, 98125 Messina, Italy; 10 Multidisciplinary Department of Medical-Surgical and Odontostomatological Specialties, University of Campania “Luigi Vanvitelli”, 80121 Naples, Italy; 11 Department of Dentistry, Faculty of Dental Sciences, University of Aldent, 1007 Tirana, Albania; 12 Clinical Microbiology, School of Dentistry, University of Modena and Reggio Emilia, 41125 Modena, Italy

**Keywords:** monkeypox, pandemic, zoonosis, oral health, dentistry

## Abstract

With the recent increased prevalence of human outbreaks, monkeypox has been recognized for decades as an infectious disease with substantial pandemic potential. The majority of cases of this virus have been observed in the European region (11,865), with few cases in the Western Pacific (54). Various governing health agencies are striving to restrain the fatal monkeypox virus (MPXV). Health practitioners around the world are learning about the many clinical manifestations of this infection, and its potential therapies. Despite the plethora of new evidence and rising cases, the essential questions remain unsolved. Thus, in this review, we have modernized the outlook for monkeypox, which will be helpful for various medical practitioners. In the light of continuing outbreaks around the world, we have also presented our assessment of the readiness of India against this outbreak, with a special focus on its effects on oral health.

## 1. Introduction

Infectious diseases are currently on the rise, including monkeypox, which is similar to smallpox/Variola virus. Monkeypox (MPX) has emerged as a global threat and has been declared a public health emergency [1]. Although less clinically severe than smallpox, monkeypox is a zoonotic disease with symptoms resembling the latter [2].

In 1958, colonies of “monkeypox” were isolated; the virus was referred to as MPX, and in 1970, the first human case was detected in the Democratic Republic of Congo [3]. Most cases of this virus are found in Central and West Africa. The first wide outbreak of monkeypox outside of Africa was documented in the United States of America in 2003 [4,5]. Monkeypox might pose a threat to developing countries, such as India. 

To date, many articles have been published on monkeypox, but few studies have discussed India’s preparedness do defend its population against this virus. India and other developing countries should approach the outbreak of monkeypox in nonendemic countries as an opportunity to bolster the capacity of their health systems and public health surveillance approaches to prepare for and respond to potential outbreaks and epidemics. With every additional case of the current outbreak, fundamental questions remain unanswered despite the abundance of new information. It is critical for healthcare professionals worldwide to update their knowledge of zoonotic infections. Thus, this paper highlights and reviews the current state of knowledge regarding MPX, its diagnosis, treatment, and prevention. This review can provide a reference for various medical personnel, including dentists, who might come across these conditions in their clinical practices. MPXV is known to have severe repercussions in young children; thus, we have briefly examined its effects in this unique population. This review also presents observations surrounding the global and Indian contexts in relation to MPX, with particular emphasis on the preparedness of India against the present outbreak, especially in relation to its implications for oral health. 

## 2. Epidemiology

### 2.1. Agent

**Figure 1:**
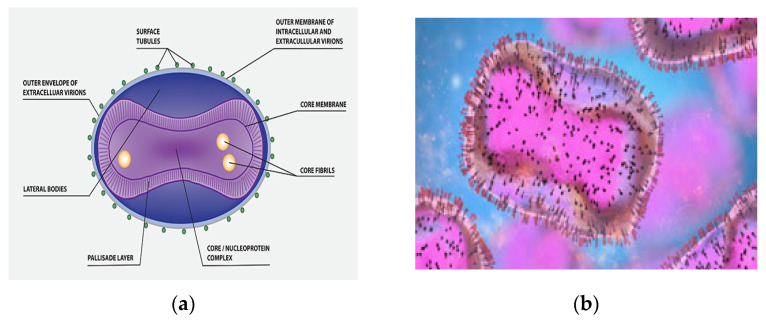
(**a**) MPXV molecular structure. (**b**) An electron micrograph displaying oval-shaped mature virus particles (pink) and immature particles (blue), taken from a sample from a patient with monkeypox.

Monkeypox virus (MPXV) belongs to the Poxviridae species [6]. In the Poxviridae family, the Orthopoxvirus gene contains two enclosed strands of virus DNA (replicating in the cytoplasm and not the nucleus) and is called the monkeypox virus (MPXV). It is an enveloped virus that is 200–250 nm length and brick-shaped (Figure 1a) [3,7].

Under an electron microscope, the monkeypox virus appears rather large with a distinctive ultrastructure with mature virus particles with shapes comparable to mulberries (Figure 1b) [8]. MPXV has a low mutation rate, and adaptive alterations of the virus may have increased its transmissibility under particular conditions. In addition, according to current investigations, MPXV in the current outbreak demonstrated single nucleotide and frameshift mutational changes in comparison with the earlier outbreaks [9].

The genomes of this virus are highly conserved middle regions which encode replication; they have changeable terminal ends containing genes which determine host range pathogenicity [10]. MPXV has a rather large genome comprising the components required for viral replication in the cytoplasm of the host cell, generating pathogenicity [11]. It has been hypothesized that the evolution of Orthopoxvirus is the consequence of increasing gene loss, especially towards the terminal end of the genome, which, combined with gene copy number variation, improves virus survival and fitness [12,13].

### 2.2. Host

Multiple investigations have indicated that this disease is also linked to animal contact. Nonhuman primates (such as squirrels, Gambian pouched rats, and dormice) are known to be naturally prone to contracting the monkeypox virus [6]. Such species include wild squirrels, pet prairie dogs, and imported rodents from Ghana [14]. Additionally, in Africa, various kinds of monkeys have been documented as additional potential animal contacts for MPXV transmission [6,15]. Small mammals, such as elephant shrews and mice, are considered to play a part in viral transmission, but the reservoir host remains unknown [16].

### 2.3. Mode of Transmission

The main way that diseases spread from one person to another is through their large airways. Droplets typically need a long period of intimate contact. It is transmissible by both direct and indirect interaction with the lesion, and interaction with body fluids, such as through sharing an infected person’s contaminated clothing or bed sheets. Viral transmission through feces may provide an additional exposure source [17]. Additionally, transmission from an infected mother to her fetus is possible [4,6,18].

Known risk factors for MPX transfer from person to person include sharing a bed, as well as practices that transfer the virus directly to oral mucosa, such as using the same fork or cup for eating or drinking. Living in an area that has recently lost its forest cover, not being vaccinated against smallpox, sleeping on the floor, and touching or eating dead bush meat or monkeys are risk factors for viral transmission in endemic areas [3,19].

Despite the fact that global reporting of MPX cases in this outbreak has asserted sexual associations, MPX is not a sexually transmitted pathogen. Recent cases have indicated a higher infection rate among males [20]. Infection is often caused by dangerous interactions with wild animals. The epidemiological and clinical standards for monkeypox are still being examined because it is unclear how widespread the disease is [21,22].

Thus, the main goals of research surrounding the virus are to understand its clinical features and complications, to provide symptom relief, and to avoid secondary infection. 

### 2.4. Current Worldwide Monkeypox Trends

The Central African clade and the West African clade are two separate genetic clades of MPXV. Historically, infection with the Central African (Congo Basin) clade is linked to high rates of transmission and cases with mortality rates of 10%, whereas infection with the West African clade has been connected to a more self-limiting condition [23].

Numerous other nations in Central and Western Africa have reported an endemic monkeypox outbreak. Additionally, some nonendemic nations such as the USA and UK have reported cases of this [7,24].

Monkeypox cases were found worldwide in 2103 instances between 1 January 2022 and 15 July 2022. Monkeypox remains a problem in 42 member states throughout five WHO regions, including America, Africa, Europe, the Eastern Mediterranean, and the Western Pacific. Since January 2022, the USA (7525), UK (2980), Spain (5162), France (2424), Germany (2980), and Brazil (2131) have all reported high numbers of confirmed cases. Additionally, eleven deaths have been attributed to monkeypox [25].

In 2022, the first death from monkeypox was reported in Nigeria. Of the reported instances, 99% involved men over 40 years of age (range: 0–65 years) [18,26].

Around 30,000 cases of monkeypox have been documented in 89 countries as of 2022; India, with a population of 1.38 billion people, is not an exemption.

### 2.5. The Route of Monkeypox to India

India recently verified two instances of monkeypox in the district of Kerala [18,27].

The first incidence of monkeypox in Southeast Asia was reported in India on July 14; the patient was a 35-year-old male who had previously visited Kerala from the United Arab Emirates (UAE). In recent months, the number of cases in India has continued to rise. India announced nine confirmed cases of monkeypox on 8 August 2022. Five of these occurred in Kerala in the southwest and four occurred in Delhi in the north; these locations are more than 2600 km apart. The Indian Council of Medical Research (ICMR) isolated a viral strain, A.2, from first two reported cases. This strain differs from the one that has affected humans in Europe [6]. Despite the increasing number of cases, the following question remains unanswered: Will MPXV cause a pandemic [28]?

### 2.6. Will It Be Referred to as the “Next COVID”?

Multiple factors mean that it is unlikely that MPXV will become a pandemic. Firstly, MPXV has existed worldwide for many years, and we have a reasonable comprehension of the virus’s composition, spread, and pathogenicity. Secondly, generally speaking, MPXV leads to moderate symptoms, as demonstrated by the low number of fatalities occurring since the pandemic began. In contrast, COVID-19 transmits via the respiratory system and has produced a large number of asymptomatic cases; thus, MPXV is less communicable because it requires close personal contact. In MPXV, an individual can transmit disease if the symptoms manifest; consequently, the likelihood of transmission occurring unnoticed is minimal [29]. Fourth, some smallpox vaccines are widely available; their “off-label” use should be advised, and global production can be scaled up as needed. Fifth, the virus is relatively stable, with a modest mutation rate. In this context, the majority of infectious disease specialists anticipate that the epidemic of monkeypox will not develop into a pandemic. Thus, we can assume that an outbreak of monkeypox can be efficiently contained by isolating confirmed cases, quarantining contacts, and using licensed smallpox vaccines “off-label” for “ring vaccination.” Currently, it is not advised to immunize large communities [30].

Hence, with the urgent increase of surveillance programs in various nonendemic nations, the support of rigorous monkeypox case findings, rapid diagnoses, and the provision of appropriate supportive care, we can easily prevent the wide transmission of this virus. More consideration must be given to populations at high risk of infection and the potential risks of monkeypox nosocomial transmission. Understanding the dynamic epidemiology of the current outbreak of monkeypox requires international cooperation, which will enable improved surveillance and contact tracing [31,32].

## 3. Clinical Features

**Figure 2:**
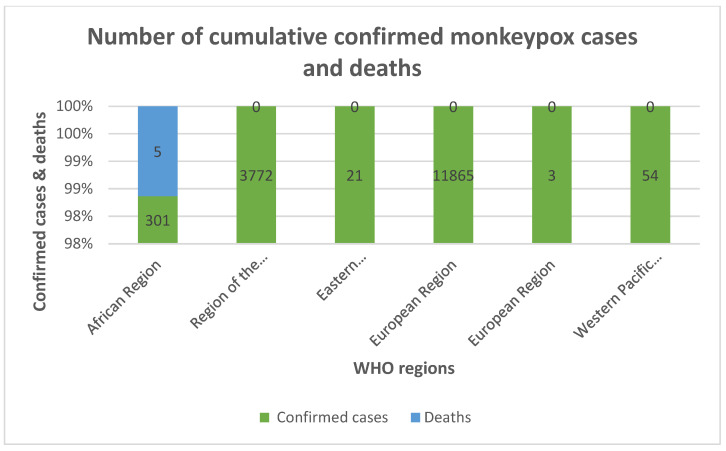
The majority of cases of this virus were observed in the European region (11,865), with few cases in the Western Pacific. The African region seemed to have the highest mortality among the regions, with 5 deaths reported due to monkeypox [27].

Three days after a smallpox-like rash appears on an MPXV-infected person’s skin, their fever goes down. The rash begins on their face (95%) and soon centrifugally transmits to the palms, and the soles of the feet (75%), the oral mucous membrane (70%), the genitals (30%), the conjunctiva, and the cornea (20%) [33]. Symptoms of monkeypox, which typically last between two and four weeks, start as macules that develop successively into papules, vesicles, pustules, and crusts (Figure 2) [5]. Fever, exhaustion, and lymphadenopathy are prodromal symptoms that continue for 2–4 days, along with some headaches, muscle aches, and back pain. In 1987, severe lymphadenopathy was the only clinical feature that distinguished MPX from varicella and smallpox [3].

Secondary infections, pneumonia, sepsis, encephalitis, and corneal involvement are examples of complications (which may lead to loss of vision). In addition, common side effects include bacterial superinfection, conjunctivitis, tonsillitis, oedema of the eyelids, hemorrhagic pustules, and pharyngitis [34,35].

Incubation period: The time frame from getting infected to the development of symptoms is typically between 6 and 13 days. The incubation period lasts for about 12 days on average, but can be up to 21 days long [4].

Historically, the case fatality ratio for monkeypox has varied between 0 and 11 percent in the general community, with the younger age group experiencing greater rates. In the recent past, the case mortality rate has typically ranged from 3 to 6 percent [4,6].

## 4. Infection Outcome and Understanding At-Risk Populations 

Monkeypox is commonly mild and self-delimiting, but children, pregnant women, patients with comorbidities, and immunocompromised hosts can develop severe cases. Miscarriages and fetal deaths have occurred as a result of monkeypox crossing the placenta. However, it is unknown whether these consequences are correlated with the severity of the maternal sickness [27].

Children are more prone to encounter severe cases, which correlate with the severity of this virus, the patient’s state, and the type of complications. In recent longitudinal research conducted in the Democratic Republic of the Congo, 216 patients suffering from monkeypox, treated between 2007 and 2021, were between the ages of 0 and 12 years. The available evidence indicates that the likelihood of children contracting the disease may have decreased over time, yet they remain a more vulnerable group due to the probability of negative effects in this demographic [26,36].

## 5. Diagnosis

Rapid diagnosis is essential for epidemic containment, but it cannot be accomplished only by observing patients clinically. Thus, laboratory confirmation is crucial since MPXV can induce illness that is clinically identical to other pox-like illnesses. WHO lists smallpox, varicella, chickenpox, measles, bacterial skin diseases, scabies, drug allergies, and syphilis among the possible differential diagnoses [37].

Polymerase chain reaction (PCR), serum immunoglobulin M and immunoglobulin G enzyme-linked immunosorbent assays, immunofluorescent antibody assays, isolating of the virus, electron microscopy, histopathological evaluation, and other tests are used to diagnose MPXV. PCR and genome sequencing are the most widely used diagnostic methods; they utilize samples from MPX lesions to validate viral presence due to its properties, such as threshold determination and viral quantification [38]. The laboratory investigation of probable MPX patients during the USA outbreak included the use of all these tests to serologically determine the presence of OPXV-specific antibodies [3]. Due to the lack of specificity in these tests, it is difficult to distinguish MPXV infection from other poxviruses [7,39]. 

The first BioThreat Alert^®^ pilot study was completed in 2012, and it yielded encouraging results using single-strain Orthopoxvirus infection specimens. This technique reliably recognized vaccinia and MPXV viruses containing 107 plaque-forming units (PFU) per milliliter and properly identified five out of the six clinical specimens that were evaluated. The results showed that it may be used as a point-of-care diagnosis for suspected MPX cases along with an effective screening tool to identify specimens that need additional tests. Although it is commercially available, BioThreat Alert^®^ is the first lateral-flow-based detection test for Orthopoxvirus. Moreover, there is no information at this time about its use in countries where MPX is endemic.

Evaluation of cases serologically can show that a person has been exposed to a virus, but it is limited in its ability to diagnose because it can also show immunological reactions to immunizations or other OPXV exposures. New, extremely sensitive immunological approaches may help with the early detection of MPX during an outbreak because research has revealed that antiviral antibodies and T-cell responses increase as the clinical symptoms of the disease begin to appear [3,40].

## 6. Oral Manifestations of MPXV

Monkeypox has been linked to herpes zoster, herpes simplex, measles, Zika, dengue, and syphilis. Small, fragile blisters on the mucosal membrane, painful aphthous ulcers, multiple scattered lesions over the skin inside the mouth and lips, facial rashes and severe pain, petechial lesions on the hard palate, and temporomandibular joint stiffness are indicative of subclinical immunosuppression associated with a monkeypox infection. It becomes very crucial for dentists to identify any oral manifestations early and follow universal precautions. Dentists should be encouraged to look out for these signs during their clinical practice in order to catch the infection in its early stages and refer the patient to a general physician for further evaluation of the case [41,42].

### 6.1. Oral Signs and Symptoms

#### 6.1.1. Lymphadenopathy

Lymph nodes are a part of the immune system and are present in various body parts including the neck, belly, armpits, groin, and mouth. Lymph nodes are lingual nodes located in the intermuscular spaces on the floor of our mouths. Infectious agents such as the monkeypox virus are also responsible for inflammation in the lymph nodes.

#### 6.1.2. Oral Lesions

Oral lesions are blisters with a spherical shape and a crimson border. It is a common symptom among people with monkeypox. The most prevalent oral scars associated with MPXV are papulopustular rashes with scarring and crust development, manifesting as progressive, vesiculopustular, desquamating, and maculopapular necrotizing dermatitis in several forms. Additionally, the formation of clefts in the interstitial spaces between cells can be observed. Later, apical progression of lesions with significant inflammation and necrosis of the superficial dermis, as well as loss of sebaceous glands, becomes evident [43].

According to the CDC, oral lesions can be noticed in about 75 percent of MPXV infections in some African regions. It affects the oral mucosa in 70% of cases, which progressively leads to vesicular formation; lips may also be affected. These lesions are well-circumscribed, circular, and deep-rooted. Some of these lesions develop umbilication, which can be seen as a sort of dot right on top of the lesion [5].

#### 6.1.3. Inflammatory Rash

Most rashes develop on the body after the end of the latent phase during a monkeypox infection. The first signs of these rashes appear on the face, and then make their way into the oral cavity [44].

Oral ulcers are tiny sores that develop in the mouth, impairing daily habits and ultimately causing parchedness (lack of moisture) and undernutrition. Initially, popular blisters and ulcerations were documented around the mouth in the outbreak. According to one study, mouth ulcers were present in nearly 23.5% of MPX patients [45,46].

In a study carried out by Thornhill et al. in 2022, 26 people demonstrated oropharyngeal symptoms such as oral or tonsillar lesions, odynophagia, epiglottitis, and pharyngitis as the first symptoms [47].

Additionally, in a study by Patel et al. in 2022, oropharyngeal lesions were seen in 13.7% of patients, while 4.6% had tonsillar erythema, pustules, or abscesses [48].

### 6.2. Precautions in Dental Clinics

Dental professionals began to follow strengthened protocols during the SARS-CoV-2 pandemic and will strive to protect patients and employees during the MPX outbreak. A patient stepping into a dental office might be in the prodromal phase. However, the first lesions to develop are often intraoral lesions. Dentists can safeguard the health and wellbeing of themselves and those in the dental office by checking the tongue of patients for any signs of redness or ulcers. This can be followed by an examination of the lymph nodes. In most cases of monkeypox virus infection, lymphadenopathy can be seen in the submandibular, cervical, axillary (armpit), and groin areas [45,49].

Dental professionals and teams should take precautions to stop the virus from spreading and provide awareness regarding droplet infections by staying in proximity. Patients are screened by keeping abreast with local cases through their regional health agencies [50].

For differential diagnosis, clinicians must be vigilant in identifying rashes mimicking MPXV and distinguish MPXV from herpetic and related vesicular–bullous lesions. Close contact with a patient’s belongings or their infected skin lesions is a major mode of virus transmission. Consequently, transmission can be minimized in dental care settings by employing universal precautions in the treatment of MPX-infected patients [45].

Because of the possibility of MPXV droplet transmission, further measures need to be implemented as determined by risk assessment. Early patient identification requires the use of adequate PPE and increased vigilance. Thus, all healthcare personnel, including dentists, should wear N95 masks, FFP3 respirators, and personal protective equipment (PPE). Patients should be examined for symptoms, common facial rashes, and intraoral lesions. The patient should be moved to a well-isolated room before providing any interventions and care should be taken to reduce exposure to other individuals, such as covering the patient’s nose and mouth with a surgical mask and covering any exposed skin sores [51,52].

## 7. Treatment

### 7.1. Antiviral Treatment

Animal trials for treating OPXV illness have been successful; no significant adverse consequences have been detected from providing antiviral treatment.

A randomized double-blinded trial was conducted in 2010, where cynomolgus macaques were given a lethal dose of MPXV. Tecovirimat (ST-246) is an antiviral chemical that inhibits seven strains of the monkeypox virus [53]. Phase I clinical trials have thus far demonstrated the safety of ST-246 as a treatment for human OPXV infection in its latent phases and as a means of disease prevention when administered during the incubation phase. The CDC advised the use of ST-246 for treatment with the onset of the MPXV epidemic in the USA. Additional investigation is required to establish whether it is effective in treating MPXV in humans [26].

In 2022, the antiviral drug Tecovirimat, originally developed for the treatment of smallpox, will be approved for the treatment of monkeypox. Tecovirimat, which was originally created to combat smallpox, is permitted for treating MPXV, but it is not yet generally available. Clinical trials on humans demonstrated that this medication can be safely administered, with very few drug reactions [54].

A retrospective observational study undertaken in the United Kingdom during 2018–2021 revealed a drop in complications, days of hospitalization, and complete coverage of the seven documented instances in different regions of the country [55].

Current therapy options include Cidofovir, a broad-spectrum antiviral medication which is active against several DNA viruses, such as MPXV [56]. When Brincidofovir (CMX001) and Cidofovir were compared in vitro, Brincidofovir demonstrated stronger adverse reactions but greater activity against the monkeypox virus. Additionally, Brincidofovir had a higher selective index [57].

Most of the treatment for monkeypox is symptomatic, including the management and prevention of complications. Thus, fluids and a proper diet are required for symptomatic relief and complete recovery. 

### 7.2. Vaccination

In 2019, the third-generation monkeypox vaccine MVA-BN (modified vaccinia Ankara–Bavarian Nordic strain) was licensed. On the basis of vaccinia virus array, this vaccination provides protection against MPXV. As of 11 June 2022, the smallpox vaccine MVA-BN is available in numerous European nations, the United States, and Nigeria, primarily for “off-label” use. Non-replicating vaccines (e.g., MVN-BN) are suggested for children and for pregnant and breastfeeding women [6,58].

Clinical trials for antiviral medicines are still ongoing, and third-generation MVA vaccines such as ACAM3000 and TBC-MVA are still being developed [3]. Smallpox vaccines are efficient in preventing postexposure prophylaxis. The JYNNEOS (Bavarian Nordic) smallpox vaccine has received approval by the US Food and Drug Administration (FDA) to prevent MPXV, and the ACAM2000 vaccination can also be effectively used [59]. ACAM2000, a live vaccination, is undergoing testing as well.

JYNNEOSTM (IMVAMUNE or Imvanex) is 85% effective in preventing both smallpox and monkeypox [60]. It is worth noting that IMVAMUNE has also been tested on health professionals in the Republic of the Congo who are at potential risk of contracting monkeypox. The FDA has licensed this vaccine to prevent monkeypox in individuals aged 18 years and older. It is important to emphasize that IMVAMUNE can be administered to individuals with AIDS or atopic dermatitis, whereas ACAM2000 cannot be administered to patients with either condition [61].

Smallpox vaccination is beneficial for MPXV prevention and postexposure prophylaxis. Nonetheless, in circumstances where smallpox vaccines are contraindicated, the vaccinia virus immunoglobulin (VIGIV) provided is similarly effective for postexposure prophylaxis. The Centers for Disease Control and Prevention’s (CDC) Expanded Access Investigational New Drug (EA-IND) protocols now permit access for using Tecovirimat, Cidofovir, and VIGIV from the Strategic National Stockpile to treat OPXV [62,63,64].

## 8. Prevention

The primary approach to preventing monkeypox infection is to increase public knowledge of risk factors and activities so that the population can minimize their risk of virus exposure. The viability and suitability of vaccination for the prevention and control of monkeypox are now being investigated scientifically. Some nations have policies in place or are in the process of implementing policies to deliver vaccines to at-risk populations, including laboratory staff, members of fast-response teams, and healthcare professionals.

### 8.1. Lowering the Risk of Human-To-Human Transmission

Prevention, together with surveillance and early detection of new infections, is critical for outbreak containment. Coming into proximity with sick people is the largest risk factor for contracting MPXV. Personnel in healthcare and household members are more vulnerable to infection.

### 8.2. Reducing the Likelihood of Zoonotic Disease Transmission

Over time, the majority of human infections have come been a result of animal-to-human transmission. Prevention approaches include advising the public to stay away from touching wild animals without protection, along with their flesh, blood, and other parts. Furthermore, all meat-related items or animal components need to be properly prepared before ingestion [4].

### 8.3. Monkeypox Prevention by Limits on Animal Trade

Some countries have put limitations on the entry of rodents and nonhuman primates to prevent monkeypox. Captive animals potentially infected with monkeypox should be promptly quarantined and isolated from other animals. All animals who may have come into contact with any infectious animal must be quarantined, treated carefully, and observed for 30 days for signs of monkeypox [6].

## 9. Preparedness and Response by India

The key to an effective reaction to a virus outbreak is stringent surveillance at a country or region’s entrance points, as well as early detection, isolation, and case management, preferably through ring vaccination (vaccination of close contacts).

The Indian Ministry of Health and Family Welfare (MoHFW) released recommendations for identification and treatment of MPXV towards the end of May 2022 [4]. Thus, India needs to prioritize outbreak prevention measures by placing emphasis on standard case definitions, heightened surveillance, and early identification of cases. India and other governments should take advantage of outbreaks in endemic-free nations to improve community health surveillance and capacity of the health system to respond to outbreaks and epidemics. The immunization expert committees and working groups of associations for professionals, as well as the National Technical Advisory Group on Immunization (NTAGI) in India, should discuss potential target populations and create technical guidelines for organizing, purchasing, stockpiling, and, if necessary, deploying such vaccines [23].

Numerous viral and zoonotic illnesses have appeared in India over the past 20 years. Global climate change is predicted to increase the risks of zoonotic diseases and the transmission of cross-species viruses. For this situation, a “One-health” strategy that combines treatments to maintain the health of people, animals, and ecosystems is necessary. The Pradhan Mantri–Ayushman Bharat Health Infrastructure Program (PM-ABHIM) was launched by the Indian government over the past year to enhance the public health laboratories and personnel [64]. If PMABHIM and PHMC are implemented quickly, then Indian states will be ready to respond to and prepare for epidemics and pandemics, which will also include detection and prevention of zoonotic infections [65].

There is an urgent need for a thorough, reliable, and long-term research plan to fully understand the monkeypox virus; to address knowledge gaps and questions about the disease’s emergence, epidemiology, and ecology; to improve surveillance capabilities; and to design suitable prevention, preparedness, and emergency response activities. Learning more about the monkeypox virus’s evolutionary genomics, shifting epidemiology, ecological niche modelling, and animal reservoirs should be prioritized, along with developing quick, evidence-based responses and management/control strategies to effectively contain outbreaks of the virus, thereby preventing human infections [66,67].

## 10. Conclusions

Monkeypox has emerged as a global threat, with an incubation period lasting an average of 12 days, with a case mortality rate of 3–6 percent. Administering antiviral medications such as Cidofovir, Brincidofovir (CMX001), and Tecovirimat (ST-246), and vaccines such as Vaccinia Immuno Globulin (VIG), has proved to be efficacious against MPXV. Standard infection control precautions have been used by dentists to deliver care for many years, and the improved processes put in place during the COVID-19 pandemic will continue to protect patients and staff during MPXV outbreaks. Dental professionals can identify the oral manifestations of MPXV, thereby helping in the early containment of this disease. Understanding the natural reservoir of viruses, with its spillover events, can help prevent the reemergence of zoonotic diseases and help in preparing against future zoonotic viruses.

Thus, this review identifies direct implications for policymakers and healthcare planners in relation to the emergence and reappearance of such viral outbreaks. This will eventually highlight the need for epidemiological awareness, investigation, and preparation. Further research should be performed to establish the research goals for zoonoses and strengthen the development of drugs and vaccines. We have already advocated interdisciplinary collaboration to combat such viral infections worldwide. Simultaneously, a deeper understanding of phylogenetics, with their shifting epidemiological patterns, is required.

## Data Availability

Not applicable.
